# Characteristics and outcomes of anti-infective de-escalation during health care-associated intra-abdominal infections

**DOI:** 10.1186/s13054-016-1267-8

**Published:** 2016-04-07

**Authors:** Philippe Montravers, Pascal Augustin, Nathalie Grall, Mathieu Desmard, Nicolas Allou, Jean-Pierre Marmuse, Jean Guglielminotti

**Affiliations:** Département d’Anesthésie Réanimation, APHP, CHU Bichat-Claude Bernard, Paris, France; Université Denis Diderot, PRESS Sorbonne Cité, Paris, France; INSERM, UMR 1137, Infection, Antimicrobiens, Modélisation, Evolution, Paris, France; Laboratoire de Microbiologie, AP-HP, CHU Bichat-Claude Bernard, Paris, France; Service de Réanimation, Centre Hospitalier Sud Francilien, Corbeil-Essonnes, France; Service de Chirurgie Générale, APHP, CHU Bichat-Claude Bernard, Paris, France

**Keywords:** De-escalation, Health care-associated intra-abdominal infections, Peritonitis, Multidrug-resistant bacteria, Antibiotic therapy

## Abstract

**Background:**

De-escalation is strongly recommended for antibiotic stewardship. No studies have addressed this issue in the context of health care-associated intra-abdominal infections (HCIAI). We analyzed the factors that could interfere with this process and their clinical consequences in intensive care unit (ICU) patients with HCIAI.

**Methods:**

All consecutive patients admitted for the management of HCIAI who survived more than 3 days following their diagnosis, who remained in the ICU for more than 3 days, and who did not undergo early reoperation during the first 3 days were analyzed prospectively in an observational, single-center study in a tertiary care university hospital.

**Results:**

Overall, 311 patients with HCIAI were admitted to the ICU. De-escalation was applied in 110 patients (53 %), and no de-escalation was reported in 96 patients (47 %) (escalation in 65 [32 %] and unchanged regimen in 31 [15 %]). Lower proportions of *Enterococcus faecium*, nonfermenting Gram-negative bacilli (NFGNB), and multidrug-resistant (MDR) strains were cultured in the de-escalation group. No clinical difference was observed at day 7 between patients who were de-escalated and those who were not. Determinants of de-escalation in multivariate analysis were adequate empiric therapy (OR 9.60, 95 % CI 4.02–22.97) and empiric use of vancomycin (OR 3.39, 95 % CI 1.46–7.87), carbapenems (OR 2.64, 95 % CI 1.01–6.91), and aminoglycosides (OR 2.31 95 % CI 1.08–4.94). The presence of NFGNB (OR 0.28, 95 % CI 0.09–0.89) and the presence of MDR bacteria (OR 0.21, 95 % CI 0.09–0.52) were risk factors for non-de-escalation. De-escalation did not change the overall duration of therapy. The risk factors for death at day 28 were presence of fungi (HR 2.64, 95 % CI 1.34–5.17), Sequential Organ Failure Assessment score on admission (HR 1.29, 95 % CI 1.16–1.42), and age (HR 1.03, 95 % CI 1.01–1.05). The survival rate expressed by a Kaplan-Meier curve was similar between groups (log-rank test *p* value 0.176).

**Conclusions:**

De-escalation is a feasible option in patients with polymicrobial infections such as HCIAI, but MDR organisms and NFGNB limit its implementation.

## Background

The modern concept of reducing both the spectrum of antimicrobial therapy and its potential to promote resistance [1], usually called *de-escalation*, is strongly recommended in all of the recently published guidelines for antibiotic stewardship [2, 3]. Several definitions have been used to describe this process [1, 4–7]. Despite the limited evidence, de-escalation has been recommended to decrease the likelihood of emergence of resistant organisms [8], to optimize activity, and to reduce toxicity and costs [3].

Two types of critically ill patients have been investigated: cohorts with a specific disease—mainly ventilator-associated pneumonia (VAP) [5, 9–13]—and large mixed populations with severe sepsis or septic shock or patients who require emergency empiric antibiotic therapy [4, 14–19]. In a recent systematic review, Tabah et al. identified isolation of multiresistant pathogens, polymicrobial infections, and intra-abdominal infections as factors negatively associated with de-escalation [20]. Only a few studies have addressed this issue in the context of intra-abdominal infections [4, 14, 18, 21, 22]. The high frequency of polymicrobial infection [23, 24] and multidrug-resistant (MDR) organisms [23, 24] as well as the presence of fungi [17, 25] in this setting might raise specific concerns, especially in health care-associated intra-abdominal infections (HCIAI).

The purpose of the present study was to analyze the frequency of de-escalation, the factors that could interfere with this process, and their clinical consequences in a cohort of intensive care unit (ICU) patients with HCIAI.

## Methods

### Study population

From January 1999 through December 2011, all consecutive patients admitted to our ICU for the management of HCIAI were prospectively included in a database and their medical charts were retrospectively reviewed. The study was approved by the local institutional review board (CEERB CHU Bichat Paris VII University, APHP, Paris, France), which waived the need for signed informed consent.

### Selection of cases and inclusion criteria

Postoperative peritonitis was defined as the first macroscopic findings of intra-abdominal infection combined with positive fluid culture yielding at least one microorganism (bacteria or fungi) at the time of reoperation (day 0) following a first abdominal surgery [24]. Several patients had to be excluded from the analysis due to early change in their clinical status before de-escalation could be instituted: patients who died during the first 3 days following surgery (microbiologic results not yet obtained), those who were discharged during the first 3 days (incapacity to adequately follow clinical outcome and antibiotic therapy), and those who underwent early reoperation during the first 3 days (high proportion of persistent infection and prolonged antibiotic therapy). Similarly, we excluded patients with negative microbiologic samples, since the concept of de-escalation is questionable and the interpretation of the results is difficult. In these patients, empiric anti-infective therapy was discontinued. Drainage of abscesses, debridement of infected and necrotic tissues, abdominal cavity cleansing, irrigation, and definitive control of the source of contamination were performed according to the surgical principles used for the management of abdominal sepsis [26]. Ostomy was preferred to primary anastomosis. No open-wound management was performed, and the abdomen was not irrigated after surgery.

### Microbiologic data

Peritoneal fluid samples were systematically collected during surgery and were immediately sent to the bacteriology laboratory [24]. Cultures were performed with identification and susceptibility testing for Gram-positive and Gram-negative aerobe bacteria, anaerobes, and fungi. Antibiotic susceptibility was determined for each organism by the disk diffusion method, according to the criteria of the Antibiogram Committee of the French Society for Microbiology [27]. MDR bacteria were defined as those resistant to three or more antimicrobial classes [28]: methicillin-resistant *Staphylococcus aureus* and methicillin-resistant coagulase-negative staphylococci; ampicillin-resistant enterococci; Enterobacteriaceae producing an extended-spectrum β-lactamase or producing a derepressed cephalosporinase; and/or nonfermenting Gram-negative bacilli (NFGNB) resistant to piperacillin-tazobactam, ceftazidime, or imipenem-cilastatin, or producing an extended-spectrum β-lactamase.

### Management of antibiotic therapy

Empiric anti-infective therapy, systematically started at day 0, took into account the severity of the case and usually combined piperacillin-tazobactam or imipenem-cilastatin with amikacin and vancomycin [23], possibly associated with antifungal therapy (mainly fluconazole) based on presumed risk factors [25, 29]. Definitive anti-infective therapy was adapted on the basis of the results of identification and antibiotic susceptibility testing (≥48 h). In both situations, therapy was considered appropriate when all cultured organisms (bacteria and fungi) were targeted. Anti-infective therapy was prescribed by the senior ICU physicians following discussion with the consultant microbiologist on a daily basis.

The following changes were considered to constitute de-escalation [1]: withdrawal of one agent (β-lactam, aminoglycoside, fluoroquinolone, vancomycin, antifungal agent) or narrowing spectrum of activity (β-lactam agents) and/or switch from combination to monotherapy. Discontinuation of unduly administered agents was also recorded. Changes among cefepime, ceftazidime, piperacillin-tazobactam, and ticarcillin-clavulanate were not considered to be significant changes of the spectrum of coverage [1].

In patients without de-escalation, two situations were identified according to previous definitions [4]. Maintained empiric treatment without modification was called unchanged therapy [4]. Escalation was defined as addition or switch to a new broad-spectrum anti-infective agent (carbapenems, glycopeptides, fluoroquinolones) [4] or upgrade to broader-spectrum β-lactams [1]. When changes combined escalation and de-escalation, the patient was assigned to the escalation group [4]. In summary, a patient receiving empiric therapy with piperacillin-tazobactam plus amikacin who was subsequently switched to piperacillin and vancomycin was classified as having withdrawal of one agent and escalation.

### Data collection

All patients’ charts were reviewed. Demographic data and severity scores (Simplified Acute Physiology Score II score [30] and Sequential Organ Failure Assessment [SOFA] score [31]) were recorded on admission to the ICU. The severity of the underlying medical condition and the presence of chronic diseases [32] were recorded. The characteristics of initial surgery were recorded.

The following clinical and severity characteristics were assessed at day 0, day 3, and day 7 after surgery for patients still in the ICU [24, 33, 34]: temperature, white blood cell count (WBC), serum creatinine, and SOFA score. Patients meeting the following three criteria at day 3 were arbitrarily defined as improving: (1) a SOFA score that decreased more than 2 points at day 3 versus day 0 or a SOFA score of 0 points, (2) a WBC that decreased more than 5000/mm^3^ between day 0 and day 3 or WBC less than 12,500/mm^3^, and (3) a temperature decrease greater than 0.5 °C between day 0 and day 3 or temperature greater than or equal to 36.5 °C and less than 38.1 °C. Similar analyses were used at day 7 to compare changes in these criteria between days 3 and 7. Medical and surgical complications, additional reoperations for persistence of the initial infection or superinfections (including MDR organisms), death between days 3 and 28 following surgery, and discharge from the hospital were assessed.

### Statistical analysis

Results are expressed as median and interquartile range (IQR) or number and proportion. Statistical significance was defined as *p* < 0.05. For statistical analysis, we used R version 2.14.1 software (R Foundation for Statistical Computing, Vienna, Austria). For comparisons between antibiotic strategy groups (de-escalation, no change, or escalation), we used the χ^2^ test and Fisher’s exact test for discrete variables and unpaired Wilcoxon tests for quantitative variables. The effect of antibiotic strategy on day 28 mortality was assessed with a Kaplan-Meier survival curve and tested with a log-rank test.

Three multivariable models were developed (1) to identify risk factors for de-escalation and (2) to assess the association between antibiotic strategy (de-escalation, no change, or escalation) and day 28 or in-hospital mortality. In univariate analysis for these three models, we used Fisher’s exact tests and Wilcoxon tests. Unadjusted ORs or HRs were calculated. Variables with a *p* value less than 0.2 in univariate analysis were entered into a multivariate logistic regression model or a Cox proportional hazards model with backward selection. For day 28 and in-hospital mortality, the antibiotic strategy was forced until the end of the selection process. Logistic models were evaluated for discrimination with the c-statistic and for calibration with the Hosmer-Lemeshow test.

## Results

### Epidemiologic and clinical characteristics

**Fig. 1:**
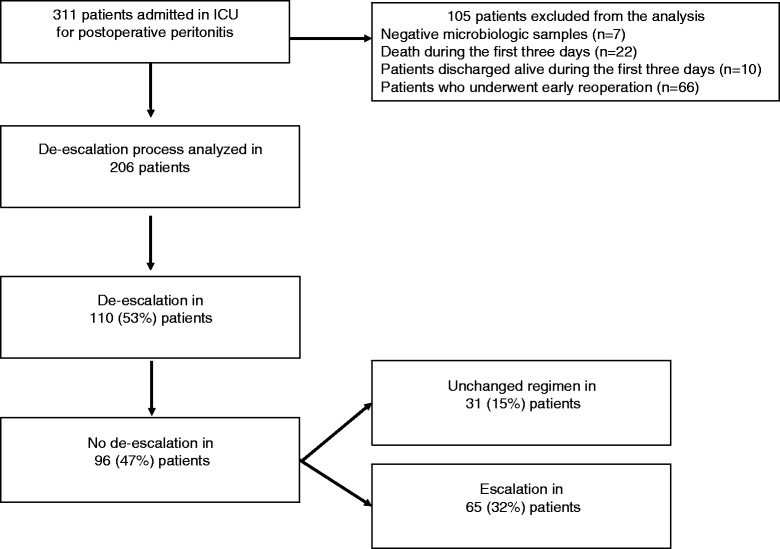
Flowchart of the 206 patients studied. *ICU* intensive care unit

During the study period, 311 ICU patients were admitted for the management of HCIAI. Figure 1 displays a flowchart of patients through the study. Overall, 105 patients were excluded, resulting in 206 patients for whom the de-escalation process was analyzed. De-escalation was performed in 110 patients (53 % of the analyzed population), and no de-escalation was observed in 96 patients (47 %) (escalation in 65 patients [32 %] and unchanged regimen in 31 patients [15 %]). De-escalation was never performed after discharge from the ICU. The frequency of de-escalation remained stable over the study period, ranging between 47 % and 63 % of the analyzed population (not significant; data not shown). Clinical characteristics were similar at day 0 in both groups (Table 1). In the non-de-escalation group, a significantly increased severity was observed in the patients with an unchanged regimen versus escalation (Table 1).

**Table 1:** Demographic and clinical characteristics of the 206 patients with or without subsequent antibiotic de-escalation

Characteristic	De-escalation (*n* = 110)	No de-escalation (*n* = 96)	Escalation (*n* = 65)	No change (*n* = 31)
Male sex, *n* (%)	61 (55)	56 (58)	35 (54)	21 (68)
Age, years, median (IQR)	61 (47–72)	66 (51–75)^a^	63 (47–75)	70 (58–77)
Comorbidities				
Fatal underlying disease	30 (27)	32 (33)	21 (32)	11 (35)
Cancer, *n* (%)	37 (34)	36 (38)	23 (35)	13 (42)
Diabetes, *n* (%)	17 (15)	15 (16)	9 (14)	6 (19)
Time since initial surgery, days, median (IQR)	7 (5–12)	7 (4–10)	6 (3–9)	8 (5–10)
Antibiotic therapy before reoperation, *n* (%)	73 (66)	68 (71)	47 (72)	21 (68)
Broad-spectrum interim antibiotic, *n* (%)	34 (31)	37 (39)	27 (42)	10 (32)
Intraoperative diagnosis				
Anastomotic leakage, *n* (%)	45 (41)	27 (28)	19 (29)	8 (26)
Perforation or ischemia, *n* (%)	33 (30)	36 (38)	20 (31)	16 (52)^a# ^
Purulent collection, *n* (%)	19 (17)	17 (18)	13 (20)	4 (13)
No cause, *n* (%)	19 (17)	20 (21)	15 (23)	5 (16)
Contamination below transverse mesocolon, *n* (%)	82 (75)	74 (77)	50 (77)	24 (77)
Characteristics at the time of ICU admission				
Bacteremia, *n* (%)	26 (24)	17 (18)	14 (22)	3 (10)
SAPS II score, median (IQR)	45 (34–54)	47 (35–57)	44 (34–56)	51 (42–61)^a# ^
SOFA score, median (IQR)	7 (4–9)	8 (4–10)	7 (4–9)	9 (6–10)
Hemodynamic failure^b^, *n* (%)	65 (59)	65 (68)	41 (63)	24 (77)
Respiratory failure^b^, *n* (%)	54 (49)	40 (42)	26 (40)	14 (45)
Renal failure^b^, *n* (%)	21 (19)	18 (19)	13 (20)	5 (16)

*IQR* interquartile range, *SAPS II* Simplified Acute Physiology Score II, *SOFA* Sequential Organ Failure Assessment

Patients without de-escalation were also analyzed in terms of subsequent antibiotic escalation or no change. Results are expressed as number and proportions or median (IQR)

^a^ *p* < 0.05 versus de-escalation

^b^SOFA score of 3 or 4 for each organ

^#^ *p* < 0.05 versus escalation

### Microbiologic analysis

Overall, 618 microorganisms from peritoneal samples were cultured (311 in patients without de-escalation, including 101 organisms in the unchanged group and 210 in the escalation group). Similar microbiologic results were observed between de-escalation and no de-escalation groups (data not shown), except for lower proportions of *Enterococcus faecium* (9 [3 %] versus 18 [7 %] without de-escalation, respectively; *p* < 0.01) and non-fermenting Gram-negative bacilli (9 [3 %] versus 22 [8 %], respectively; *p* < 0.01). Among patients without de-escalation, increased proportions of Gram-negative bacteria and *Enterobacteriaceae* were observed in patients with unchanged regimen compared with those whose regimen was escalated (47 [49 %] versus 69 [37 %], respectively [*p* < 0.05]; and 38 [40 %] versus 52 [28 %] [*p* < 0.05]). In the de-escalation group, 23 (21 %) of 110 patients harbored MDR strains compared with 54 (56 %) of 96 patients in the non-de-escalation group (*p* < 0.01) (Table 2).

**Table 2:** Multidrug-resistant bacteria cultured from peritoneal fluid of patients with or without subsequent antibiotic de-escalation

Microorganisms	De-escalation	No de-escalation	Escalation	No change
Total number of multidrug-resistant bacteria, *n* (%)	29 (9)	74 (24)^a^	58 (28)	16 (16)^b^
Gram-positive bacteria, *n* (%)	15 (5)	39 (13)^a^	33 (16)	6 (6)^b^
Enterococci, *n* (%)	3 (1)	9 (3)	7 (3)	2 (2)
Staphylococci, *n* (%)	12 (4)	29 (9)	25 (12)	4 (4)
*Staphylococcus aureus*, *n* (%)	–	8 (3)	6 (3)	2 (2)
Gram-negative bacteria, *n* (%)	14 (5)	35 (11)^a^	25 (12)	10 (10)
*Enterobacteriaceae*, *n* (%)	10 (3)	24 (8)	18 (9)	6 (6)
*Escherichia coli*, *n* (%)	1 (0)	11 (4)	8 (4)	3 (3)
*Enterobacter* spp., *n* (%)	5 (2)	9 (3)	8 (4)	1 (1)
Nonfermenting Gram-negative bacilli, *n* (%)	3 (1)	11 (4)	7 (3)	4 (4)
*Pseudomonas* spp., *n* (%)	2 (1)	6 (2)	4 (2)	2 (2)
Total number of cultured bacteria, *n*	307	311	210	101

Among the 96 patients without de-escalation, the results were analyzed in terms of subsequent antibiotic escalation or no change

^a^ *p* < 0.01 versus de-escalation

^b^ *p* < 0.05 versus escalation

### Anti-infective therapy

De-escalation was performed on a microbiologic basis in a median delay of 3 days (IQR 2–4) after surgery. Empiric treatments and the procedures applied for de-escalation at day 3 are described in Table 3. Empiric use of combination therapy, carbapenems, glycopeptides, and antifungal agents were significantly more frequent in the de-escalation group. Among antifungal agents, echinocandins were minimally prescribed for empiric therapy (six patients in the de-escalation group with discontinuation in all but one case and one switch to azoles, one patient in the non-de-escalation group).

**Table 3:** Anti-infective regimens in patients with or without de-escalation and clinical characteristics at day 3

	De-escalation (*n* = 110)	No de-escalation (*n* = 96)	Escalation (*n* = 65)	No change (*n* = 31)
Empiric antibiotic therapy				
Monotherapy, *n* (%)	13 (12)	32 (33)^a^	20 (31)	12 (29)
Combination of two drugs, *n* (%)	40 (36)	34 (35)	26 (40)	8 (26)
Combination of three drugs or more, *n* (%)	57 (49)	30 (33)^b^	19 (29)	11 (35)
Carbapenem, *n* (%)	35 (32)	15 (16)^a^	10 (15)	5 (16)
Piperacillin-tazobactam, *n* (%)	67 (61)	60 (63)	40 (62)	20 (65)
Vancomycin, *n* (%)	57 (52)	23 (24)^a^	15 (23)	8 (26)
Aminoglycosides, *n* (%)	59 (54)	33 (34)^a^	27 (42)	6 (19)^b^
Fluoroquinolones, *n* (%)	6 (5)	11 (11)	10 (15)	1 (3)
Antifungal therapy, *n* (%)	47 (43)	23 (24)^a^	11 (17)	12 (39)^b^
Azoles, *n* (%)	41 (37)	20 (21)^a^	9 (14)	11 (35)^b^
Adequate empiric therapy, *n* (%)	100 (91)	37 (39)^a^	9 (14)	28 (90)^c^
Reevaluation of antibiotic therapy				
Discontinuation of carbapenems^d^, *n* (%)	27/35 (77)	4/15 (27)	4/10 (40)	–
Discontinuation of piperacillin-tazobactam^d^, *n* (%)	50/67 (75)	25/60 (42)	25/40 (63)	–
Discontinuation of vancomycin^d^, *n* (%)	46/57 (81)	6/23 (26)	6/15 (40)	–
Discontinuation of aminoglycosides^d^, *n* (%)	54/59 (92)	21/33 (64)	21/27 (78)	–
Discontinuation of fluoroquinolones^d^, *n* (%)	2/6 (33)	6/11 (55)	6/10 (60)	–
Discontinuation of antifungal agent ^d^, *n* (%)	23/47 (49)	4/23 (17)	4/11 (36)	–
Withdrawal of at least one agent, *n* (%)	110 (100)	42 (47)^a^	42 (65)	–
Narrowing spectrum, *n* (%)	74 (67)	18 (19)^a^	18 (28)	–
Switch to monotherapy, *n* (%)	54 (49)	7 (7)^a^	7 (11)	–
Interruption of unnecessary agent, *n* (%)	78 (71)	20 (21)^a^	20 (31)	–
Clinical changes between days 0 and 3				
Changes in SOFA score, median (IQR)	−2 (−4 to -1)	−2 (−4 to 0)	−2 (−4 to 0)	−2 (−3 to 0)
Decreased SOFA score, *n* (%)	69 (63)	57 (59)	38 (58)	19 (61)
Decreased temperature, *n* (%)	69 (63)	64 (67)	41 (63)	23 (74)
Decreased WBC, *n* (%)	38 (35)	32 (33)	23 (35)	9 (29)
Clinical improvement at day 3, *n* (%)	17 (15)	18 (19)	14 (22)	4 (13)

*IQR* interquartile range, *SOFA* Sequential Organ Failure Assessment, *WBC* white blood cell count

Among those without de-escalation, the results were analyzed in terms of subsequent antibiotic escalation or no change. Results are expressed as number and proportion of the total number of patients

^a^ *p* < 0.01 versus de-escalation

^b^ *p* < 0.05 versus de-escalation

^c^ *p* < 0.01 versus escalation therapy

^d^Proportions are expressed as the number of discontinuations of the drug to the total number of patients empirically receiving this class of drug

When taking into account the criteria for de-escalation, we found that 33 patients met three criteria (withdrawing, narrowing, and switching) in the de-escalation group but none of those who did not de-escalate. However, two criteria (withdrawing and narrowing) were reported in 17 patients in the non-de-escalation group. No clinical change between days 0 and 3 allowed patients who were subsequently de-escalated to be differentiated from those who did not (Table 3).

Determinants of de-escalation in multivariate analysis were adequate empiric therapy (OR 9.60, 95 % CI 4.02–22.97, *p* < 0.001), empiric use of vancomycin (OR 3.39, 95 % CI 1.46–7.87, *p* = 0.004), carbapenems (OR 2.64, 95 % CI 1.01–6.91, *p* = 0.04), and aminoglycosides (OR 2.31, 95 % CI 1.08–4.94, *p* = 0.03), while presence of NFGNB (OR 0.28, 95 % CI 0.09–0.89, *p* = 0.03) and presence of MDR bacteria (OR 0.21, 95 % CI 0.09–0.52, *p* < 0.001) were the risk factors for non-de-escalation (c-index 0.880, 95 % CI 0.832–0.928, Hosmer-Lemeshow test *p* = 0.14) (Table 4).

**Table 4:** Uni- and multivariate analyses of risk factors for de-escalation

	Univariate analysis				Multivariate analysis	
	De-escalation (*n* = 110)	No de-escalation (*n* = 96)	Unadjusted odds ratio (95 % CI)	*p* value^a^	Adjusted odds ratio (95 % CI)	*p* value
At time of admission						
Age, years	61 (47–72)	66 (51–75)	0.98 (0.97–0.99)	0.049	–	–
Emergency surgery	37 (34)	44 (46)	0.60 (0.34–1.05)	0.087		
Anastomotic leakage	45 (41)	27 (28)	1.76 (0.98–3.17)	0.058	–	–
Empiric antibiotic monotherapy	13 (12)	32 (33)	0.26 (0.13–0.54)	<0.01	–	–
Initial use of carbapenems	35 (32)	15 (16)	2.52 (1.27–4.98)	0.009	2.64 (1.01–6.91)	0.047
Initial use of vancomycin	57 (52)	23 (24)	3.41 (1.87–6.21)	<0.0001	3.39 (1.46–7.87)	0.004
Initial use of aminoglycosides	59 (54)	33 (34)	2.20 (1.25–3.88)	0.007	2.31 (1.08–4.94)	0.031
Initial use of fluoroquinolones	6 (5)	11 (11)	0.44 (0.15–1.22)	0.134		
Initial use of antifungal agents	47 (43)	23 (24)	2.36 (1.29–4.32)	0.005	–	–
At day 3						
Presence of *Enterococcus faecium*	8 (7)	18 (19)	0.33 (0.14–0.82)	0.019	–	–
Presence of streptococci	31 (28)	17 (18)	1.82 (0.93–3.55)	0.098	–	–
Presence of staphylococci	25 (23)	35 (36)	0.51 (0.27–0.94)	0.032	–	–
Presence of NFGNB	8 (7)	22 (23)	0.26 (0.11–0.62)	0.002	0.28 (0.09–0.89)	0.031
Presence of MDR strains	23 (21)	54 (56)	0.20 (0.11– 0.37)	<0.0001	0.21 (0.09–0.52)	0.0007
Presence of fungi	30 (27)	41 (43)	0.50 (0.28–0.90)	0.027	–	–
Adequate empiric therapy	100 (91)	37 (39)	15.95 (7.38–34.40)	<0.0001	9.60 (4.02–22.97)	<0.0001

*MDR* multidrug-resistant, *NFGNB* nonfermenting Gram-negative bacilli

The c-index of the final model is 0.880 (95 % CI 0.832–0.928), and the Hosmer-Lemeshow test *p* value is 0.14

^a^ *p* values are derived from Fisher’s exact test or Wilcoxon test

At day 3, no difference was observed between patients with an unchanged regimen and those who underwent escalation, except for the higher proportions of aminoglycosides and antifungal therapy and the very low proportion adequate empiric therapy (Table 3).

### Clinical evaluation following de-escalation

When comparing the patients who were de-escalated and those who did not de-escalate, we found no significant difference between days 3 and 7 (Table 5). In addition, no significant difference in morbidity or mortality criteria was observed between these two groups.

**Table 5:** Clinical presentation at day 7 of empiric therapy and outcome in patients with or without de-escalation

	De-escalation (*n* = 110)	No de-escalation (*n* = 96)	Escalation (*n* = 65)	No change (*n* = 31)
Definitive anti-infective therapy				
Monotherapy, *n* (%)	64 (58)	21 (22)	9 (14)	12(29)^a^
Combination of two drugs, *n* (%)	33 (30)	39 (41)	31 (48)	8 (26)^b^
Combination of three drugs or more, *n* (%)	13 (12)	36 (38)	25 (38)	11 (35)
Use of carbapenems, *n* (%)	5 (5)	23 (24)^a^	18 (28)	5 (16)
Use of piperacillin-tazobactam, *n* (%)	28 (25)	39 (41)^b^	19 (29)	20 (65)^a^
Use of vancomycin, *n* (%)	11 (10)	41 (43)^a^	33 (51)	8 (26)^b^
Use of antifungals, *n* (%)	29 (26)	43 (45)^a^	31 (48)	12 (39)
Use of azoles, *n* (%)	28 (25)	39 (41)^b^	28 (43)	11 (35)
Use of echinocandins, *n* (%)	1 (1)	3 (3)	3 (5)	–
Duration of anti-infective therapy, days, median (IQR)	10 (10–14)	10 (10–14)	10 (10–14)	10 (10–14)
Clinical changes between days 3 and 7				
Number of cases at day 7	91	75	51	24
Changes in SOFA score^c^, median (IQR)	−1 (−3 to 0)	−2 (−4 to 0)	−2 (−4 to 0)	−2 (−4 to 0)
Decreased SOFA score^c^, *n* (%)	57 (63)	48 (64)	35 (54)	13 (42)
Decreased temperature^c^, *n* (%)	38 (42)	36 (48)	27 (42)	9 (29)
Decreased WBC^c^, *n* (%)	31 (34)	17 (23)	12 (18)	5 (16)
Clinical improvement at day 7^c^, *n* (%)	16 (18)	9 (12)	9 (14)	–
Discharge between days 3 and 7, *n* (%)	20 (22)	15 (20)	10 (20)	5 (21)
Death between days 3 and 7, *n* (%)	1 (1)	3 (4)	2 (4)	1 (4)
Medical complications	12 (12)	14 (16)	6 (10)	8 (29)
Surgical complications	26 (25)	21 (24)	10 (17)	11 (39)^b^
Reoperation, *n* (%)	38 (35)	35 (36)	19 (29)	16 (51)^b^
Time to reoperation, days, median (IQR)	6 (5–9)	6 (5–8)	5 (4–8)	7 (6–10)
Superinfection on subsequent reoperation^d^, *n* (%)	23 (61)	23 (66)	13 (68)	10 (63)
Emergence of MDR strains^d^, *n* (%)	21 (55)	20 (57)	11 (58)	9 (56)
Emergence of ESBL *Enterobacteriaceae* ^d^, *n* (%)	5 (13)	5 (14)	3 (16)	2 (13)
Emergence of MDR NFGNB^d^, *n* (%)	9 (24)	5 (14)	2 (11)	3 (19)
Emergence of MRSA^d^, *n* (%)	9 (24)	8 (23)	6 (32)	2 (13)
Duration of mechanical ventilation^e^, days, median (IQR)	7 (3–13)	7 (3–11)	7 (2–10)	7 (3–15)
ICU length of stay^e^, days, median (IQR)	12 (8–20)	12 (8–21)	12 (8–21)	14 (5–23)
Survival at day 28, *n* (%)	91 (83)	72 (75)	51 (78)	21 (68)
ICU mortality rate, *n* (%)	23 (21)	32 (33)^b^	18 (28)	14 (45)
Hospital mortality rate, *n* (%)	25 (23)	33 (34)	19 (29)	14 (45)

*ESBL* extended-spectrum β-lactamase, *ICU* intensive care unit, *IQR* interquartile range, *MDR* multidrug-resistant, MRSA methicillin-resistant *Staphylococcus aureus*, *NFGNB* nonfermenting Gram-negative bacilli, *SOFA* Sequential Organ Failure Assessment, *WBC* white blood cell count

Among those without de-escalation, the results were analyzed in terms of subsequent antibiotic escalation or no change

^a^ *p* < 0.01 versus escalation therapy

^b^ *p* < 0.05 versus escalation therapy

^c^Results expressed as number of patients at day 7 in the same group

^d^Results expressed as number of patients who underwent reoperation in the same group

^e^Results calculated for ICU survivor patients

**Fig. 2:**
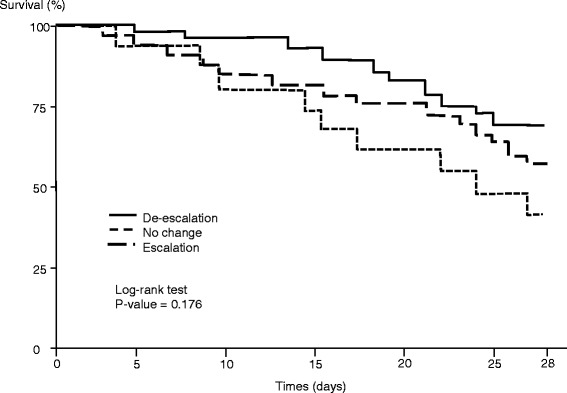
Kaplan-Meier survival curves of patients with de-escalation, without any change, and with escalation

No significant differences in morbidity or mortality criteria were observed between patients who underwent escalation and those with an unchanged regimen, except for significantly increased proportions of surgical complications and reoperations in patients with an unchanged regimen. The risk factors for death at day 28 following surgery in a Cox model are presented in Table 6. The survival rate expressed by a Kaplan-Meier curve was similar between groups (log-rank test *p* value 0.176) (Fig. 2).

**Table 6:** Uni- and multivariate analyses of risk factors for 28-day mortality

	Univariate analysis				Multivariate analysis	
	Death at day 28 (*n* = 43)	Survival at day 28 (*n* = 163)	Unadjusted hazard ratio (95 % CI)	*p* value^a^	Adjusted hazard ratio (95 % CI)	*p* value
Age, years	69 (56–78)	62 (46–72)	1.022 (1.003–1.042)	0.023	1.034 (1.011–1.059)	0.004
Emergency surgery	24 (56)	57 (35)	2.072 (1.135–3.783)	0.015	–	–
Surgery below the mesocolon	28 (65)	128 (79)	0.561 (0.299–1.050)	0.075	0.427 (0.215–0.848)	0.015
SOFA score	10 (7–11)	7 (4–9)	1.261 (1.153–1.380)	<0.0001	1.291 (1.168–1.427)	<0.0001
SAPS II score	52 (45–61)	44 (32–53)	1.039 (1.019–1.060)	<0.0001	–	–
Initial use of piperacillin-tazobactam	22 (51)	105 (64)	0.636 (0.350–1.157)	0.117	–	–
Empiric antifungal therapy	21 (49)	49 (30)	1.980 (1.089–3.601)	0.029	–	–
Other *Enterobacteriaceae* ^b^	5 (12)	34 (21)	0.538 (0.212–1.366)	0.195	0.342 (0.1219–0.961)	0.0419
Presence of *Candida* spp.	19 (44)	52 (32)	1.569 (0.859–2.865)	0.150	2.641 (1.3471–5.179)	0.0047
Decreased SOFA score at day 3	17 (40)	109 (67)	0.372 (0.202–0.686)	0.0015	0.311 (0.1632–0.593)	0.0004
Antibiotic strategy				0.189		
De-escalation	19 (44)	91 (56)	0.488 (0.227–1.051)		0.566 (0.2503–1.278)	0.171
No change	10 (23)	21 (13)	Reference		Reference	Reference
Escalation	14 (33)	51 (31)	0.627 (0.278–1.411)		0.508 (0.2154–1.198)	0.122

*SAPS II* Simplified Acute Physiology Score II, *SOFA* Sequential Organ Failure Assessment

^a^ *p* values are from Fisher exact tests or Wilcoxon tests

^b^Other Enterobacteriaceae: analysis of all *Enterobacteriaceae* except *Escherichia coli*, *Klebsiella* spp., and *Enterobacter* spp.

The risk factors of in-hospital mortality on multivariate analysis were the emergency initial surgery (OR 2.81, 95 % CI 1.30–6.05, *p* = 0.008) and SOFA score on admission (OR 1.42, 95 % CI 1.24–1.62, *p* < 0.001), while a decreased SOFA score at day 3 had a protective value (OR 0.14, 95 % CI 0.06–0.31, *p* < 0.001) (c-index 0.852, 95 % CI 0.794–0.910, Hosmer-Lemeshow test *p* = 0.28).

## Discussion

In this single-center observational study, de-escalation was performed in 53 % of patients treated for HCIAI. De-escalation concerned both antibacterial and antifungal therapies. The de-escalation procedure did not modify outcome. No initial clinical characteristic allowed identification of patients who were subsequently de-escalated. The presence of MDR bacteria and NFGNB as well as initial monotherapy were the most relevant factors limiting de-escalation. No emergence of resistant organisms was observed following de-escalation in the patients who underwent subsequent reoperation.

In the absence of data in the literature, we consider that our results provide an encouraging perspective for antibiotic de-escalation in ICU patients with abdominal sepsis. However, some limitations should be considered. Only 110 (35 %) of 311 patients treated for HCIAI were de-escalated. This highly selected population and the inclusion and exclusion criteria could be considered to constitute a weakness, but they allow selection of cases in which de-escalation is possible. Our local policy for empiric and definitive antibiotic use and the local characteristics of microbial flora must be considered cautiously and cannot be generalized. The long study duration may also have led to changes in the case mix or in antibiotic susceptibility patterns over time, although our analysis did not confirm this hypothesis. Another major limitation in the interpretation of our results is the lack of assessment of the quality of source control. The exclusion of patients undergoing early reoperation probably limited the importance of this issue. The absence of consensual definitions for de-escalation is another issue to be considered. The quality of de-escalation could be considered incomplete in many patients in whom there is room for improvement, and further reduction of antibiotic use or the use of narrow-spectrum empiric therapy should be considered.

Observational and retrospective studies have suggested that the de-escalation strategy is a safe approach in patients with severe sepsis or septic shock [10–12, 15, 18, 19, 22, 35]. In recent European observational trials, the de-escalation rate ranged between 12.8 % of cases in a multicenter study in 41 French ICUs [35] and 64 % in a single-center analysis focused on patients with severe sepsis and septic shock [15]. Some prospective observational studies have suggested that mortality rates were at least not worse than those observed in patients not de-escalated [16, 36–38]. In these reports, the lengths of ICU and hospital stay were not significantly different [36, 37]. Other authors have reported that de-escalation therapy could even significantly improve the prognosis [4, 13, 39].

Prospective randomized trials addressing the issue of de-escalation are extremely rare. In a cohort of 290 patients treated for VAP, Micek et al. reported a decreased duration of antibiotic therapy and no significant differences in terms of secondary episodes of VAP and hospital mortality [37]. In a group of 116 patients with severe sepsis either assigned or not to de-escalation, Leone et al. demonstrated that de-escalation was inferior to continuation of the initial antibiotic therapy with length of stay as the primary outcome parameter [40]. Furthermore, antibiotic use was higher in the de-escalation group with a higher number of superinfections in the de-escalation group, but mortality was similar in the two groups [40].

Only four retrospective, single-center, observational studies have described de-escalation practices for patients with peritonitis [14, 18, 21, 22], including between 113 and 229 patients, 10–38 % of whom presented with peritonitis. Although the observed de-escalation rate was 23–58 % in this population, none of these studies provided any information on the outcome of de-escalated patients. Our analysis is the first to focus on this surgical population, and our results suggest that de-escalation is safe and does not change the clinical outcome. On the basis of our results, a prospective multicenter study would appear to be feasible.

Obstacles to de-escalation have been clearly identified in the literature [20]. The lack of appropriate empiric therapy is the first point to be considered [13, 22]. A high rate of MDR bacteria is an obvious reason for inadequate empiric therapy and consequently a recognized factor for limited de-escalation [20]. However, although a recent analysis suggested that polymicrobial infection was a negative factor for de-escalation [20], we did not observe this trend in the patients in our present study. Narrow-spectrum empiric therapy is obviously another important determinant limiting the frequency of de-escalation [22]. However, narrow-spectrum empiric therapy is not an issue in intra-abdominal infections in which treatment should at least target anaerobes and Enterobacteriaceae [41]. On the contrary, monotherapy has been proposed for the treatment of peritonitis [41], and this policy could be a limitation on de-escalation [18].

Poor or absent clinical improvement is another factor limiting de-escalation. In the present study, clinical and laboratory parameters of day 3 were unable to differentiate patients in whom de-escalation would be feasible and those in whom de-escalation could not be performed. Interestingly, Garnacho-Montero et al. reported lower SOFA scores at the time of de-escalation [4], suggesting that the criteria for de-escalation may change from one population to another. This also means that prescribers should rely on microbiologic samples and the laboratory results more than any other criteria. The confidence of prescribers in their initial therapy is also based on two parameters that have been only minimally assessed in the literature: adequacy of source control and pharmacokinetics of anti-infective agents.

Several reports have indicated early improvement in patients who underwent de-escalation. Paskovaty et al., in a cohort of adult patients with cancer admitted to the ICU for severe sepsis, reported a significantly decreased SOFA score on day 5 [42]. Two studies of patients with nosocomial pneumonia and ICU-acquired pneumonia reported early decreased SOFA and Acute Physiology and Chronic Health Evaluation II scores in the de-escalation groups [43, 44]. On the contrary, the incidence of organ failure at day 7 was similar in our patients with or without de-escalation. There is no obvious explanation for this discrepancy, but medical and surgical patients with sepsis may respond in different ways.

The rate of antibiotic escalation, although regularly discussed, is rarely assessed in the literature. In recent papers, this rate has ranged between 6.6 % and 7.9 % [18, 22]. However, Garnacho et al. reported escalation in 19 % of patients despite adequate empiric therapy [4]. The mortality rate in this cohort was significantly increased compared with de-escalation or unchanged therapy (42.9 % versus 27.4 % and 32.6 %, respectively; *p* = 0.006) [4], while Gonzales et al. reported that escalation did not induce any significant change in prognosis [22]. In these two studies, the heterogeneous case mix resulted in complex analysis of these data. Few studies have reported the frequency and prognosis of escalation in peritonitis, but the effect of escalation appears to be less obvious in the present cohort.

Several beneficial effects of de-escalation have been hypothesized, including preservation of the patient’s ecology and decreased emergence of MDR pathogens [3, 45]. However, these assumptions have never been clearly demonstrated. In the present study, we did not observe any significant change in the emergence of resistant pathogens in either the intra-abdominal site or extra-abdominal sites following de-escalation. This is not surprising, as the detection of emerging MDR organisms was not a specific goal of this study and changes of gut microbiota of our patients were not targeted. Similarly, de-escalation does not change the overall duration of therapy. This point, already mentioned in other studies [22], was also observed in our present study.

An abundant literature exists regarding assessment of antibiotic de-escalation, but few data are available for antifungal agents. Several reports in candidemia or invasive candidiasis suggest that antifungal de-escalation is feasible [46–48], but no study has specifically addressed the issue of intra-abdominal infections. Unwarranted antifungal prescription is frequently reported in ICU patients, which raises both ecological and financial concerns [49]. De-escalation and/or discontinuation of antifungal treatments could be proposed more frequently. Our data suggest that antifungal de-escalation could be feasible with no specific complications.

## Conclusions

De-escalation is a reasonable option, even in patients with polymicrobial infections such as HCIAI. However, MDR bacteria and NFGNB remain major obstacles to implementation of de-escalation. The prescriber must consider whether the determinants of success have been met, especially an adequate empiric therapy. Although our results are reassuring, this strategy needs to be confirmed in a multicenter, randomized, prospective trial.

## Key messages

- De-escalation is a reasonable option, even in polymicrobial infections such as health care-associated intra-abdominal infections.
- Multidrug-resistant bacteria and nonfermenting Gram-negative bacilli remain a major obstacle in de-escalation.
- The key determinants for de-escalation are susceptible microorganisms and adequate empiric therapy.

## Abbreviations

ESBL: extended-spectrum β-lactamase
HCIAI: health care–associated intra-abdominal infection
ICU: intensive care unit
IQR: interquartile range
MDR: multidrug-resistant
MRSA: methicillin-resistant *Staphylococcus aureus*
NFGNB: nonfermenting Gram-negative bacilli
SAPS II: Simplified Acute Physiology Score II
SOFA: Sequential Organ Failure Assessment
VAP: ventilator-associated pneumonia
WBC: white blood cell count
